# Transrenal Ureteral Embolization Utilizing Amplatzer Vascular Plugs and N-Butyl Cyanoacrylate Glue

**DOI:** 10.1089/cren.2018.0027

**Published:** 2018-07-01

**Authors:** Michael X. Jin, Anthony D. Mohabir, Drew M. Caplin, Igor Lobko, David N. Siegel

**Affiliations:** ^1^ Donald and Barbara Zucker School of Medicine at Hofstra Northwell, Hempstead, New York.; ^2^ Department of Interventional Radiology, Long Island Jewish Hospital, Queens, New York.; ^3^ Donald and Barbara Zucker School of Medicine at Hofstra Northwell, Hempstead, New York.

**Keywords:** ureteral embolization, Amplatzer vascular plugs, cystitis, vesicular fistulas, bladder carcinoma

## Abstract

This retrospective study presents three consecutive patients who underwent bilateral ureteral occlusion using the Amplatzer vascular plugs and N-butyl cyanoacrylate glue sandwich method. The patients were 63- and 65-year-old males and a 79-year-old female. Indications for the procedure included severe cystitis and complex vesicular fistulas unresponsive to urinary diversion. All three patients had immediate resolution of urinary leakage, resulting in symptom relief throughout the follow-up period. There were no procedure-related complications or side effects.

## Introduction

Radiation cystitis, vesicular fistulas, and bladder carcinoma are associated with significant reductions in patients' quality of life and often result in pain, embarrassment from urinary leakage, and significant patient morbidity.^1^ Conservative treatment with pain medication and urinary diversion is often inadequate in addressing symptoms for these patients.^2,3^ In addition, many of these patients are poor candidates for a major surgical reconstruction due to age, comorbidities, and body habitus.^2,3^

In the management of patients with severe bladder pathologies, ureteral embolization can be used to prevent urine from reaching the bladder, reducing discomfort from bladder irritation and leakage from fistulas.^1,2^ Embolization, which is intended to be permanent, would only be considered in patients who are not candidates for surgical reconstruction and with symptoms refractory to long-term nephrostomy drainage alone or in combination with other treatments. Agents that have been utilized for ureteral embolization include liquid embolics, coils, balloons, and sponges.^1–3^ More recently, combinations of embolization agents have been used, taking advantage of the strengths of individual agents to create a fast-setting but permanent occlusion. One novel technique utilizes an NBCA-Amplatzer “sandwich,” a combination of N-butyl cyanoacrylate (NBCA, TRUFILL; DePuy Synthes, West Chester, PA) injected between two Amplatzer vascular plugs (St. Jude Medical, St. Paul, MN) to create an immediate but long-lasting result.^1,4^ Although this method was first described in 2013, there are still limited data on the effectiveness and potential consequences of this technique.

Our report describes the use of the NBCA-Amplatzer “sandwich” technique for ureteral occlusion in three patients: two patients with severe, chronic cystitis and one patient with a vesicovaginal fistula. Outcomes and embolization stability are evaluated at 1-month follow-up.

## Methods

Three consecutive patients who underwent bilateral ureteral embolization between January 2017 and October 2017 are included: 63- and 65-year-old males and a 79-year-old female. Indications for embolization include severe pain and hematuria for the two male patients and a vesicovaginal fistula in the female patient. Bilateral embolization was performed in all three patients for a total of six ureteral occlusions. Each of four ureters was occluded with two Amplatzer vascular plugs and 1 cc NBCA per ureter sandwiched in between. Each of two ureters of the 65-year-old male was occluded using three Amplatzer vascular plugs with 1 cc NBCA per ureter sandwiched in between the two plugs distal to the kidneys due to mild reflux of NBCA glue into the proximal ureter. Since the use of Amplatzer plug and NBCA glue was for an off-label application and therefore lacked official guidelines, plug sizing and placement location within the ureter were left to the discretion of the operator.

**FIG. 1.**
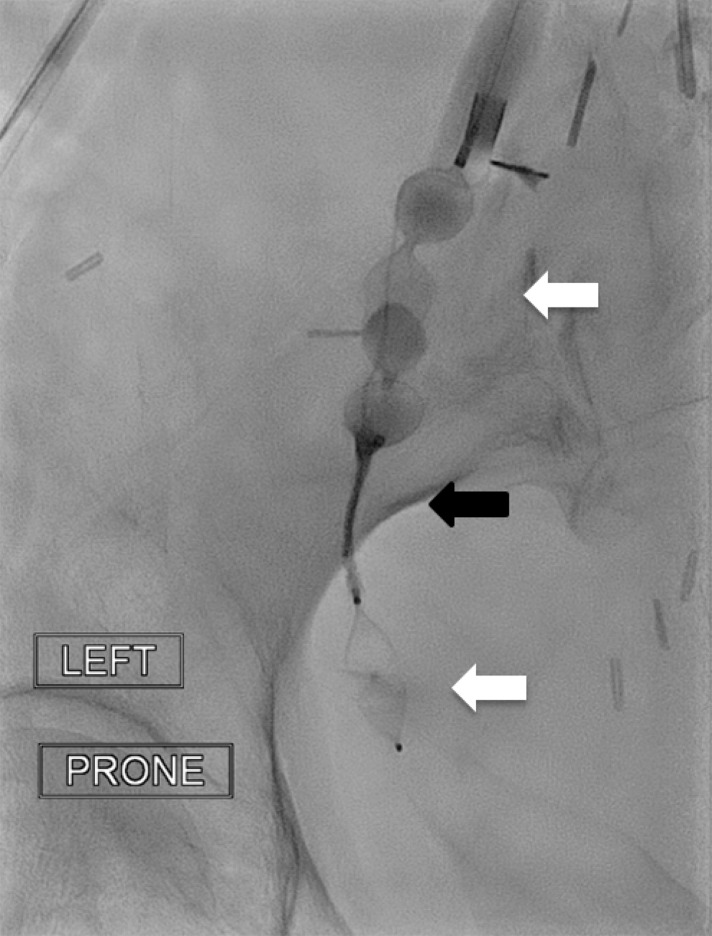
Fluoroscopy image from a 79-year-old woman who presented with persistent urinary leakage from a vesicovaginal fistula. After insertion of the sheath into the ureter, the first plug is deployed. A microcatheter is positioned proximal to the first plug, and a second plug placed proximal to the microcatheter tip. The NBCA/Lipiodol glue is then injected between the two plugs. Image shows ureteral embolization with the sandwich technique NBCA glue injection via microcatheter (*black arrow*) between two plugs (*white arrows*).

**FIG. 2.**
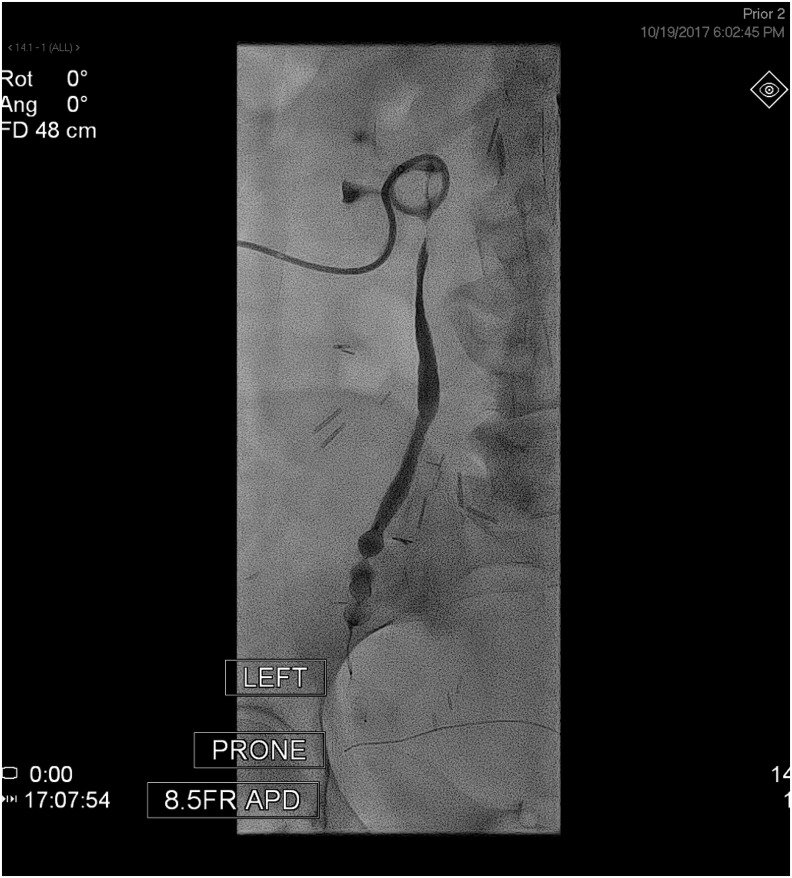
Antegrade pyelogram demonstrating contrast flow to the level of the proximal vascular plug without evidence of flow distally.

Using an 8F vascular sheath, a 12-mm plug was placed into the distal ureter via access obtained through an existing percutaneous nephrostomy tract. For each patient, contrast material was first injected in a pre-existing nephrostomy tube. The nephrostomy was then exchanged over a stiff guidewire for an 8F sheath. A second guidewire was used as a safety wire and inserted through the sheath into the renal pelvis. The sheath was then reinserted over the working wire and advanced to the mid ureter. The first Amplatzer plug was then deployed through the sheath. A 5F angled end-hole catheter was then inserted through the sheath. and a microcatheter was then inserted through the 5F diagnostic catheter and used to inject 0.5 to 1.0 mL of a 2:3 mixture of NBCA glue and Lipiodol (Ultra Fluide GMP; Guerbet, Roissy, France) into the ureter proximal to the first plug. The microcatheter and diagnostic 5F catheter were removed, and a second plug was placed via the sheath in the ureter immediately proximal to the glue (Fig. 1). For patients RE and EB, the initial 8 mm plug was placed immediately above the pelvic brim with a 12 mm plug placed ∼5 mm proximally to the first plug. 0.7 cc of 40% NBCA glue was used for RE and 0.5 cc of 40% NBCA glue was used for EB. For patient EM, an initial 12 mm plug was placed immediately below the pelvic brim and a 12 mm plug placed ∼5 mm proximally to the first plug. An additional 12 mm plug was placed in the right ureter and 8 mm plug was placed in the left ureter to prevent potential glue backflow. Forty percent NBCA glue, 1.0 cc, was used in between the distal two plugs (Fig. 2).

At the procedure's conclusion, all patients had a percutaneous nephrostomy tube placed into the renal pelvis for urinary drainage. Technical success in this study was measured as effective placement of the occlusion plugs and injection of NBCA acrylic with embolization of the ureter confirmed on antegrade pyelogram. Clinical success measurements include resolution of pain, hematuria, and urine leakage for the respective patients.

## Results

**FIG. 3.**
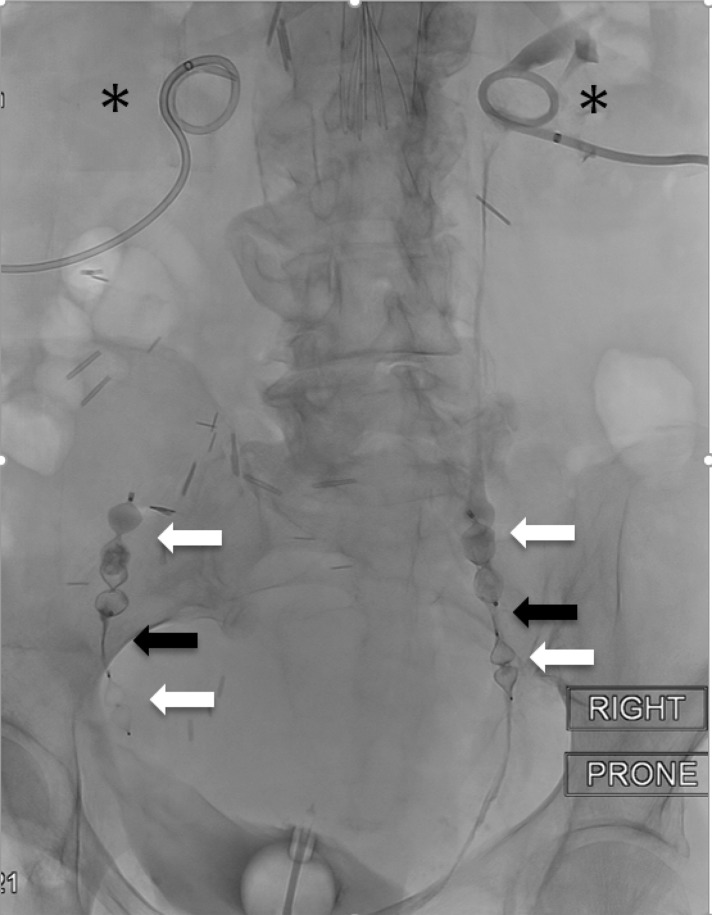
Final fluoroscopic image demonstrates effective bilateral ureteral occlusion using the “sandwich” method. Vascular plugs (*white arrows*), opaque NBCA glue (*black arrows*), and bilateral nephrostomy tubes (*asterisks*).

Three patients underwent bilateral ureter embolization in an 11-month period. Two plugs were used per ureter in two patients with one patient receiving three plugs per ureter to prevent backflow of glue to the renal pelvic. All three patients underwent embolization in the interventional radiology department and remained in the hospital for observation for at least 3 days. Immediate technical and clinical success was achieved for all patients with no urinary leakage or migration of embolic agents (Fig. 3).

The plug diameter used ranged from 8 to 12 mm and on average 0.733 cc of NCBA was used between the three patients with a range of 0.5 to 1 cc. All three patients report alleviation of symptoms. Patients report no ureteral embolization-related complications; in addition, there was no clinical evidence of ureteral recanalization at 1-month follow-up for any of the patients.

## Discussion

Ureteral embolization is a minimally invasive method technique for management of patients with chronic, severe cystitis or complex vesicular fistulas. This procedure is a valued option in patients who have failed more conservative management and are not candidates for cystectomy. There currently exists multiple agents for ureteral embolization such as cyanoacrylate, balloons, and gelatin sponge, which often demonstrate immediate occlusion but are prone to migration and/or recanalization over time.^1,2^ Other agents such as coils or metallic plugs usually result in longer term occlusion by causing urothelial scarring; however, this effect often takes weeks to develop.^3^ The Amplatzer vascular plug and NBCA sandwich method take advantage of the immediate embolization effects of the NBCA and the long-lasting effects of the Amplatzer vascular plug to create an immediate but long-lasting solution for permanent occlusion of ureters. Similar “sandwich” methods have been used in the past using a gelatin sponge nested within a coil ball for permanent ureteral occlusion; however, migration of embolic into the renal pelvis and nephrostomy catheter occlusion requiring replacement have been reported.^4^ These two complications have not been observed with the Amplatzer vascular plug and NBCA sandwich method in the current or past studies.

Limitations to this study include the small sample size and the nonexperimental nature of reported cases. Due to the off-label use of the Amplatzer plug and the NBCA glue and Lipiodol mixture, there were no guidelines on appropriate glue to Lipiodol ratio, volume of administered liquid embolic, and sizing of plugs within the ureter. Plug size selection and quantity were instead left to the clinical judgment of the operator. In addition, further investigation needs to be undertaken to identify the optimal location of plug placement and the appropriate degree of oversizing to prevent plug slippage due to peristalsis of ureters. Extensive oversizing is not recommended as anecdotal evidence suggests adjacent tissue may be damaged. This effect was seen in a previous study, in which the formation of adjacent iliac artery pseudoaneurysms was observed.^4^ Future studies could investigate the optimal ratio of plug to ureter size for ureteral embolization in animal models to ensure optimal placement of plugs. Finally, it is recommended that patients undergoing this procedure have bilateral nephrostomy tube exchanges at regular intervals due to risk of infection, dislodgement, and obstruction of the tubes. However, since patients for whom this treatment is being considered are not surgical candidates and suffer severe morbidity from their underlying conditions, this treatment may improve their overall quality of life despite continued nephrostomy tube exchanges. Thus, intervention should be decided on a case to case basis.

## Conclusion

The NBCA-Amplatzer “sandwich” method is a promising nonsurgical technique for immediate and permanent ureteral occlusion for the treatment of several entities, including vesicovaginal fistulas and chronic, severe cystitis. Our study adds three more patients to the literature who have undergone this procedure with both clinical and technical success.

## Abbreviation Used

NBCA: N-butyl cyanoacrylate
